# Decrease of mRNA Editing after Spinal Cord Injury is Caused by Down-regulation of ADAR2 that is Triggered by Inflammatory Response

**DOI:** 10.1038/srep12615

**Published:** 2015-07-30

**Authors:** Antonio Fabio Di Narzo, Alexey Kozlenkov, Yongchao Ge, Bin Zhang, Leo Sanelli, Zacnicte May, Yanqing Li, Karim Fouad, Christopher Cardozo, Eugene V Koonin, David J Bennett, Stella Dracheva

**Affiliations:** 1 Department of Genetics and Genomic Sciences, Icahn School of Medicine at Mount Sinai, New York, NY, USA; 2 The Friedman Brain Institute and Department of Psychiatry, Icahn School of Medicine at Mount Sinai, New York, NY, USA; 3 James J. Peters VA Medical Center, Bronx, NY; 4 Department of Neurology, Icahn School of Medicine at Mount Sinai, New York, NY, USA; 5 Centre for Neuroscience, Faculty of Rehabilitation Medicine, University of Alberta, Edmonton, Alberta, Canada; 6 National Center for Biotechnology Information, National Library of Medicine, National Institutes of Health, Bethesda, MD, USA

## Abstract

We recently showed that spinal cord injury (SCI) leads to a decrease in mRNA editing of serotonin receptor 2C (5-HT_2C_R) contributing to post-SCI spasticity. Here we study post-SCI mRNA editing and global gene expression using massively parallel sequencing. Evidence is presented that the decrease in 5-HT_2C_R editing is caused by down-regulation of adenosine deaminase ADAR2 and that editing of at least one other ADAR2 target, potassium channel Kv1.1, is decreased after SCI. Bayesian network analysis of genome-wide transcriptome data indicates that down-regulation of ADAR2 (1) is triggered by persistent inflammatory response to SCI that is associated with activation of microglia and (2) results in changes in neuronal gene expression that are likely to contribute both to post-SCI restoration of neuronal excitability and muscle spasms. These findings have broad implications for other diseases of the Central Nervous System and could open new avenues for developing efficacious antispastic treatments.

Severe Spinal Cord Injury (SCI) causes an immediate, devastating loss of motor function. This effect is triggered not only by damage to brain-derived axons that use fast glutamatergic synaptic transmission and facilitate voluntary initiation of movement, but also by loss of descending brainstem tracts which provide the spinal cord with its major source of neuromodulators such as serotonin (5-HT)^1^,^2^. Many of the neurons that coordinate rhythmic locomotor movements in mammals are located in the spinal cord. By activating specific receptors, serotonin facilitates voltage-gated persistent inward currents (PICs) of Ca^2+^ and Na^+^ in these spinal motoneurons, thus setting them into an excitable state^3^. Because of their unusually low threshold, the PICs are easily activated by brief synaptic inputs and play a crucial role in amplifying and prolonging the action of these inputs, ultimately enabling sustained muscle contractions^4^. When SCI eliminates brainstem-derived serotonin, motoneurons are left in an unexcitable state with small PICs, consistent with the flaccid paralysis and areflexia that are observed early after SCI^5^,^6^.

Surprisingly, despite the continued absence of serotonin, over the weeks after SCI, rudimentary locomotor-like movements spontaneously emerge, coinciding with recovery of motoneuron excitability, large PICs and associated sustained firing^7^. Unlike before injury, however, the powerful depolarizing action of PICs is difficult to terminate, because motoneurons have weaker inhibitory inputs from spinal interneurons that are themselves regulated by descending tracts^6^,^8^. Thus, in both humans^9^ and rats^4^,^6^, the PICs can lead to excessive motoneuron activity that produces uncontrolled and debilitating muscle spasms, which are readily triggered by SCI-enhanced synaptic inputs arising from normally innocuous cutaneous stimulation or muscle stretch^10^. The biological basis for the post-SCI recovery of spinal motoneuron activity remains unknown; however, recent studies have suggested that the serotonin receptors 2C (5-HT_2C_R) and 2A (5-HT_2A_R) on motoneurons become constitutively active (i.e., active in the absence of serotonin) to compensate for the loss of the brainstem neurotransmitter, ultimately facilitating the recovery of motoneuron excitability and related motor function, but also contributing to muscle spasms^11^,^12^.

5-HT_2A/2C_Rs are two highly similar G-protein-coupled receptors that exhibit considerable constitutive activity and mostly signal by activating phospholipase C (PLC)-associated cascade^13^. Whereas the constitutive activity of 5-HT_2A_R is determined by the level of its expression, 5-HT_2C_R is regulated by editing of its pre-mRNA via adenosine-to-inosine (A-to-I) deamination catalyzed by RNA-specific adenosine deaminases (ADAR1 and ADAR2)^14^,^15^,^16^. In mammals, site-specific mRNA editing resulting in re-coding is known to occur in only a few transcripts that mostly encode CNS-expressed proteins involved in neurotransmission, including 5-HT_2C_R, several glutamate receptor subunits, α3 subunit of GABA_A_ receptor, voltage-gated potassium (Kv1.1) and calcium (Cav1.3) channels^17^,^18^. Notably, the editing occurs at functionally critical positions of these proteins and hence substantially affects neuronal excitability and signal transduction.

The 5-HT_2C_R mRNA can be edited at up to five closely-spaced (within a 15 nucleotide sequence) adenine positions (denoted A, B, C, D and E sites). Because inosine is decoded as guanosine during translation, editing can alter codons for three amino acids in the second intracellular loop of the receptor^19^,^20^, a region involved in the coupling with G-proteins^21^. Combinatorial editing at the five sites can generate up to 32 mRNA variants encoding 24 receptor isoforms (sites A and B as well as sites E and C are in the same codons). The extent of editing is inversely correlated with the 5-HT_2C_R activity such that the more highly edited isoforms are less active than less extensively edited ones^16^. The unedited Ile156-Asn158-Ile160 (INI) isoform possesses considerable constitutive and agonist-stimulated activity. Conversely, when the mRNA is edited, the coupling of 5-HT_2C_R to G-proteins and its affinity for serotonin are drastically reduced. For example, compared to the INI isoform, the VSV (Val156-Ser158-Val160) isoform that can be encoded by two different mRNA variants, namely *ACD* (edited at sites A, C, and D) or *ABCD* (edited at sites A, B, C, and D), shows a 4-to-5-fold decreased coupling to signaling pathways and 4-fold reduced constitutive activity^19^,^22^.

Our recent study that used a chronic spinal rat model of spasticity^5^ has suggested that, months after sacrospinal transection, there is an alteration in 5-HT_2C_R mRNA editing which leads to increased expression of constitutively active receptor isoforms coinciding with restoration of large Ca^2+^ PICs in the motoneurons^11^. Blocking constitutively active 5-HT_2C_Rs with inverse agonists attenuates these Ca^2+^ currents as well as muscle spasms^11^. This remarkable adaptation of motoneurons via editing potentially opens new possibilities for antispastic drug therapy.

The present report aimed to characterize the nature and the extent of RNA editing alterations after SCI and to elucidate the molecular pathways that trigger and follow these editing alterations. We present evidence that the post-SCI decrease of 5-HT_2C_R editing is due to down-regulation of ADAR2 (but not ADAR1) expression, and that SCI triggers editing changes in at least one additional ADAR2 target, namely Kv1.1 channel. To elucidate the pathways that are related to alteration of mRNA editing following SCI, we examine the genome-wide transcriptional response to SCI using RNA-seq in conjunction with sophisticated bioinformatic approaches, including weighted gene co-expression network and Bayesian network analyses.

## Results

### 5-HT_2C_R Editing Is Altered in the Chronic Spinal Rat Model of Spasticity

**Figure 1:**
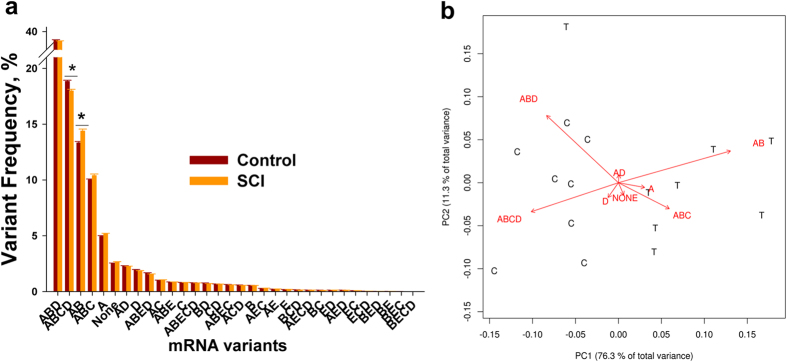
Analysis of 5-HT_2C_R editing in the spinal cord. (**a**) Shown are Means ± SE of the 5-HT_2C_R editing variant frequencies in the spinal cord of the SCI and control rats (N = 8 rats per group) obtained by MPS. *indicates a significant difference between the groups (see Fig. 2A for details). (**b**) PCA of the data in (**a**). Shown are first two PCs of all 5-HT_2C_R editing variants; *ABCD* and *AB* variants are aligned along the first principal component, which by itself explains ~ 76.3% of the total data variability. T - treatment (SCI), C - control.

We performed a detailed assessment of the SCI-induced alterations in 5-HT_2C_R mRNA editing in spinal cord samples obtained below the site of injury from sacrospinal (8 weeks post-SCI) and sham-operated (control) rats (N = 8 per group) using massively parallel sequencing (MPS) of PCR-amplified DNA fragments in the region of editing^23^,^24^. On average, 1,300,000 ± 80,000 (Mean ± SE) reads were obtained for each sample, enabling a reliable detection of all 32 possible mRNA variants (including the rarest ones^25^) in each sample (Fig. 1a). The frequencies of the variants within the sacral region were similar to those detected in the thoracic cord^23^: the *ABD* variant (edited at sites A, B, and D) accounted for ~36%, *ABCD* ~18%, and *AB* (edited at A, and B) ~14%, whereas the unedited version (*NONE)* accounted for only ~3% of all 5-HT_2C_R transcripts. Only five variants, *ABD, ABCD, AB, ABC,* and *A,* were observed at >5% frequencies. The principal component analysis (PCA) of the 5-HT_2C_R variants showed that the *ABCD* and *AB* aligned along the first PC, which by itself explained 76.3% of the total data variance (Fig. 1b). With the exception of one outlier, the SCI and control animals were perfectly segregated along the first PC, with SCI rats having lower values of *ABCD* and higher values of *AB* compared to controls.

**Figure 2:**
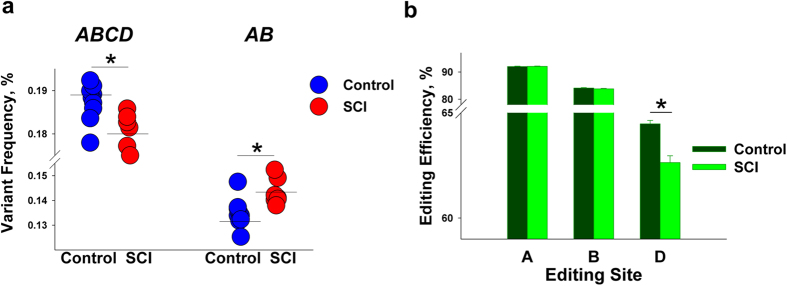
Alteration of 5-HT_2C_R RNA editing after SCI. (**a**) 5-HT_2C_R mRNA variants: *ABCD* is decreased and *AB* is increased in SCI rats (p-values < 3.2e-4). Mean frequency values in each group are shown as horizontal lines. (**b**) 5-HT_2C_R editing sites: Editing efficiency is decreased at site D in SCI rats (p = 2e-4). (**a,b**) N = 8 per group; *indicates a significant difference between the groups.

We found that the frequency of *ABCD* (which encodes a low-functioning receptor isoform VSV) was significantly lower, and the frequency of *AB* (which encodes a high-functioning isoform VNI) was significantly higher in the SCI vs. control rats (Figs 1a and 2a). We also found that, although there were no differences in editing efficiencies at the A or B as well as at the E or C sites (not-shown), significantly lower editing efficiency was observed at the D site of the SCI rats (Fig. 2b). Thus, SCI leads to a decrease in 5-HT_2C_R mRNA editing below the site of injury that is mostly accounted for by the lower editing level at the D site.

### SCI Induces Alterations of ADAR2 Expression and K_v_1.1 mRNA Editing

**Figure 3:**
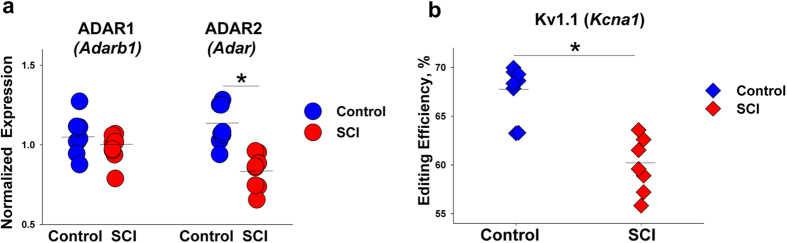
Alterations of ADARs’ mRNA expression and Kv.1.1 mRNA editing after SCI. (**a**) ADAR2 (encoded by *Adarb1*) mRNA expression was decreased (p = 2.2E-4) and ADAR1 (encoded by *Adar*) mRNA expression did not differ between SCI vs. control rats. The measurements were performed by qPCR. Expression values were normalized to geometric mean of 3 endogenous control genes—*Gapdh, Actb,* and *Ppia*. (**b**) Kv1.1 (encoded by *Kcna1*) mRNA editing is decreased in SCI vs. control rats (p = 3.3E-4). Measurements of *Kcna1* editing were performed using *Mfe1* restriction assay (see Methods). (**a,b**) N = 8 per group; *indicates a significant difference between the groups. Mean expression (**a**) or editing efficiency (**b**) values in each group are shown as horizontal lines.

In 5-HT_2C_R, sites A and B are predominantly edited by ADAR1 whereas site D is mostly edited by ADAR2^16^. Therefore, we investigated whether the observed SCI-induced editing alterations correlate with changes in the expression of editing enzymes. In line with our finding of decreased editing at the D site, but not at the A or B sites, we detected significantly lower expression of *Adarb1* (that encodes ADAR2) but not of *Adar* (that encodes ADAR1) in the SCI rats (Fig. 3a).

We next tested if SCI triggers editing changes in another protein, K_v_1.1 channel encoded by *Kcna1*, which could contribute to the recovery of motoneuron excitability and the concurrent spasticity. The K_v_1.1 mRNA is edited by ADAR2 at a single site resulting in a Val-to-Ile substitution^26^. We detected a significant decrease of the *Kcna1* mRNA editing in the SCI rats (Fig. 3b). Thus, in addition to 5-HT_2C_R, SCI induces editing changes in at least one other protein whose mRNA is edited by ADAR2 and that plays an important role in neurotransmission.

### SCI Induces Alteration of 5-HT_2A_R Expression

**Figure 4:**
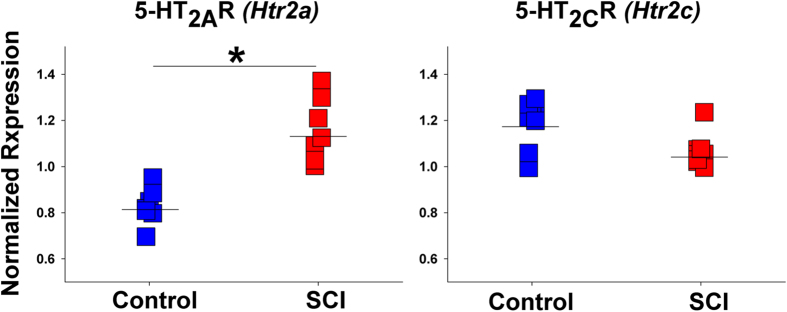
Expression of 5-HT_2A_R and 5-HT_2C_R following SCI. 5-HT_2A_R (encoded by *Htr2a*) mRNA expression was increased (p = 4.6e-5) and 5-HT_2C_R (encoded by *Htr2c*) mRNA expression did not differ between SCI vs. control rats. N = 8 rats per group. The measurements were performed by qPCR as in Fig. 3. *indicates a significant difference between the groups. Mean expression values in each group are shown as horizontal lines.

Because 5-HT2_A_R mRNA does not undergo editing, its constitutive activity is mostly determined by the expression level. Using qPCR, we investigated if mRNA expression of 5-HT_2C_R or 5-HT_2A_R (encoded by *Htr2c* and *Htr2a*, respectively) is influenced by SCI. Whereas there were no differences in the expression of *Htr2c* (which represented the total expression of all 5-HT_2C_R mRNA variants), we detected significantly higher expression of *Htr2a* in SCI animals (Fig. 4). This finding was in line with the results of previous studies which reported post-SCI up-regulation of 5-HT_2A_R mRNA and protein^12^,^23^.

### Global Transcriptional Alterations Following SCI

**Figure 5:**
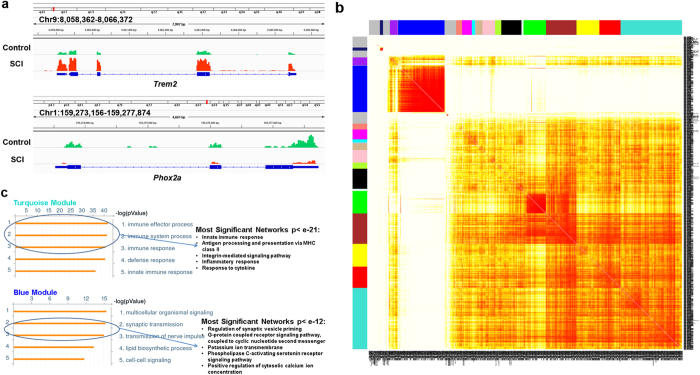
Transcriptional alterations following SCI. (**a**) The RNA-Seq data in the vicinity of loci for *Trem2* and *Phox2a*. The traces of RNA abundance show a significantly higher expression of *Trem2* and significantly lower expression of *Phox2a* in SCI vs. control rats. For visualization, the data were uploaded to Integrative Genomics Viewer (IGV) visualization tool^57^. (**b**) WGCNA analysis of DE genes. Clustering of the DE genes by topological overlap reveals 17 gene modules that are characterized by distinct expression patterns comprised of highly co-regulated genes. The color intensity in the heatmap is proportional to the interaction strength between two genes with red color for the strongest interaction and white color for no interaction. The 17 modules are represented by the color bars along the X- and Y-axes. (**C**) Functional enrichment for genes within Turquoise and Blue Modules shown in (**b**). Depicted are five most significant GO Processes identified by MetaCore that are enriched within the Turquoise and Blue modules and most significant networks within several GO processes (encircled).

We then assessed post-SCI transcriptional alterations genome-wide, using RNA-seq of the same RNA preparations that were used in the analysis of editing described above. A total of 14,638 genes were found to be expressed at a detectable level in all animals. Among these genes, 981 were differentially expressed (DE) between SCI vs. control rats: 245 transcripts were down-regulated and 736 were up-regulated (see Supplementary Table1 and examples in Fig. 5a). Although not significant after the adjustment for multiple comparison, expression of *Adarb1* was decreased (adjusted p = 0.06) and expression of *Htr2a* was increased (adjusted p = 0.1) in the SCI animals, compatible with our qPCR data.

Because of the large number of the DE transcripts, we next applied weighted gene co-expression network analysis (WGCNA) to characterize functional organization of the SCI-induced transcriptional alterations^27^. WGCNA identifies putative biologically relevant changes in gene expression by grouping genes into modules with strongly covarying patterns across the samples. Consequently, WGCNA can distinguish gene expression networks associated with specific cell types (e.g., neurons, oligodendrocytes, astrocytes, or microglia) that are present in a heterogeneous sample (e.g., brain or spinal cord) thanks to the distinct transcriptional profiles of these cell types and variation in their relative proportions across the samples^28^. The gene co-expression modules can be examined for cell-type specificity using cell type-enriched gene sets identified in previous studies^29^,^30^,^31^. WGCNA also enables the identification of modules of functionally related, highly coexpressed genes within different cellular populations^28^, and has been successfully employed to identify novel pathways and target genes for complex human diseases such as cancer, Alzheimer’s disease, obesity and diabetes^32^,^33^,^34^.

Among the DE genes, we identified 17 co-expression modules, 8 of which included more than 50 genes (Fig. 5b, Supplementary Table2). We examined the two major modules (Turquoise and Blue), which contained 197 and 148 DE genes, respectively. The majority of the Turquoise Module genes (189 of 197) were up-regulated whereas the majority of the Blue Module genes (144 from 148) were down-regulated following SCI (Bonferroni corrected enrichment p-values < 1e-100). Examination of functional annotations using GeneGo MetaCore software revealed that the Turquoise Module was significantly enriched in genes related to immunity, probably reflecting the SCI-induced inflammatory response, whereas the Blue Module was enriched in genes related to neurotransmission and lipid metabolism (Fig. 5c; Supplementary Table3). More detailed analysis revealed several networks associated with neurotransmission-related Gene Ontology categories including *potassium ion transport*, *PLC-activating serotonin receptor signaling pathway, and positive regulation of cytosolic calcium ion concentration* (all p values < 4.0 × 10^−12^) (Fig. 5c).

We then explored cell-specificity of the WGCNA modules by measuring the overlap between the module genes and cell type-specific gene signatures for neurons, oligodendrocytes, astrocytes, and microglia from the Allen Brain Atlas (http://www.brain-map.org/). The signature of neuronal and oligodendrocyte genes was significantly enriched only in the Blue Module, whereas microglia and astrocyte genes were enriched in multiple modules (Turquoise, Brown, Grey, Yellow, Green, and Red) (Supplementary Table4).

In summary, transcriptional changes following SCI clearly revealed at least two distinct components. The first component is related to the long-lasting inflammatory response (that is still present 8 weeks post-SCI) and is enriched in microglia- and astrocyte-specific genes. The second component is related to post-SCI dysregulation of neurotransmission and is represented by genes that are mostly expressed in neurons and oligodendrocytes.

### Persistent spinal microgliosis 8 weeks post-SCI

**Figure 6:**
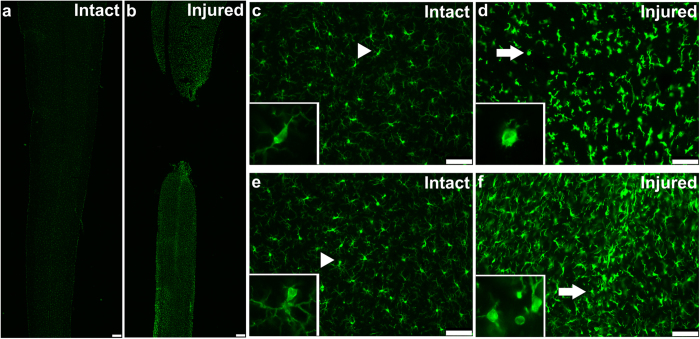
Persistent spinal microgliosis 8 weeks post-SCI. Fluorescence microscope images of longitudinal spinal cord tissue sections in a control (intact) (**a,c,e**) and an SCI (injured) rat 8 weeks after complete transection injury (**b,d,f**). In the SCI rats, the images were taken 0.6 mm above and below injury. Sections were stained with Iba-1 (green). (**a**) and (**b**) were taken at 50x magnification and encompass an entire section through the middle of the spinal cord, near the central canal. (**c**–**f**) were taken at 200x magnification in the grey matter. Microglia in the intact spinal cord (**c,e**) have elaborate thin processes which extend in various directions (arrow heads). Note increased staining intensity, rounder cell bodies and shorter and thicker processes (arrows) rostral (**d**) and caudal (**f**) to the site of injury. Insets show microglia cell morphology at 400x magnification. Scale bar (**a,b**), 250 μm. Scale bar (**c**–**f**), 75 μm.

We then assessed the involvement of neuroinflammation in the observed transcriptional alterations using Iba-1 immunohistochemistry in the sacral spinal cord 8 weeks after SCI. Iba-1 expression is up-regulated upon activation of microglia due to inflammation; Iba-1 is also expressed by infiltrating macrophages. We detected increased staining intensity, rounder cell bodies, and shorter and thicker processes in SCI rats both rostral and caudal to the site of injury, which is indicative of substantial microgliosis (Fig. 6). Notably, the entire caudal cord expressed similar gliosis, whereas the rostral cord showed gliosis up to 2.6 mm above the injury. Also, caudal to the injury, the enhanced Iba-1 expression was not changed for at least 6 months (longest point tested) after SCI, whereas rostral to the injury, normal Iba-1 expression was detected at 6 months (not-shown). Approximately, twice as many Iba-1 expressing cells were found rostral and caudal to the injury compared to the intact spinal cord. However, we could not determine from these data whether the cells were resident microglia or infiltrating macrophages from the periphery.

### Association between 5-HT2CR editing and genome-wide gene expression

**Figure 7:**
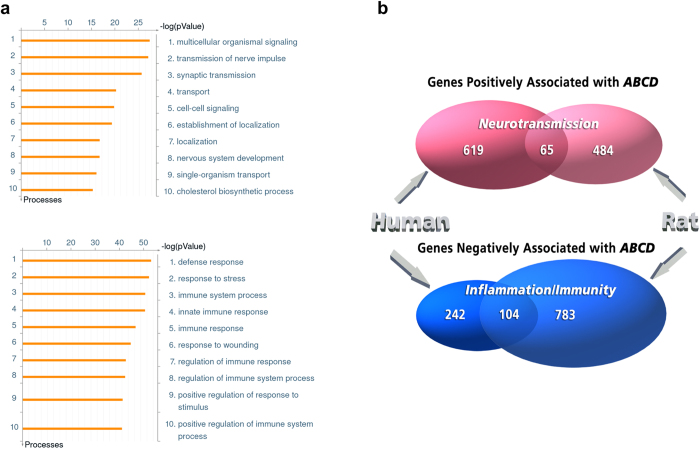
Analysis of association between 5-HT_2C_R editing and genome-wide gene expression. (**a**) Functional enrichment for genes that are negatively (upper panel) or positively (lower panel) associated with *ABCD*. Depicted are ten most significant GO Processes identified by MetaCore for each list of genes. (**b**) Venn diagrams depicting the overlap among genes that are positively or negatively associated with *ABCD* in the human PFC^35^ and in the rat model of SCI (this study). Shown are the numbers of genes as well as GO categories of enrichment.

To investigate the pathways that are linked to 5-HT_2C_R editing, we performed an analysis of the association between editing and genome-wide gene expression using the RNA-seq data obtained from SCI and control rats (N = 16). Because >75% of the total variability of 5-HT_2C_R editing was explained by PC1 (dominated by the *ABCD* vs. *AB* axis) (Fig. 1), we used the frequency of the *ABCD* variant (hereinafter *“ABCD*”) as a proxy of the editing level in each animal. Among the 14,638 genes detected in all samples, expression of 1,436 genes showed a significant correlation with *ABCD* (FDR ≤ 0.1): 549 genes were positively and 887 were negatively correlated (Supplementary Table5). Functional annotation analysis of the positively associated genes revealed significant enrichment in genes implicated in transmission of nerve impulse, synaptic transmission and cholesterol biosynthesis (FDR < 1e-12), whereas negatively associated genes showed significant enrichment for genes involved in stress and immune response (FDR < 1e-50), (Fig. 7a and Supplementary Table6). Expression of *Adarb1*, but not *Adar*, was positively associated with *ABCD*.

We have recently reported an analysis of the association between genome-wide gene expression and *ABCD* in the autopsy specimens of the human prefrontal cortex (PFC)^35^. We identified 684 genes whose expression was positively and 346 genes whose expression was negatively associated with *ABCD* (positive and negative *ABCD* signatures, respectively). Similar to the present report, the positive signature was enriched in genes involved in synaptic transmission, whereas the negative signature was enriched in genes involved in inflammation and immunity. Also similar to the present study in rats, expression of *ADARB1* (encodes ADAR2), but not *ADAR* (encodes ADAR1) was positively associated with *ABCD* in the human data set^35^.

Notably, 11,997 genes were expressed in both human PFC and rat spinal cord. We observed a significant overlap between the genes that were positively (N = 65; odds ratio = 3.34, 95% C.I: 2.49–4.42, p = 2.6e-14) or negatively (N = 104; odds ratio = 8.55, 95% C.I.: 6.59–11.05, p < 1e-15) associated with *ABCD* in both data sets (Fig. 7b Supplementary Table7). Functional annotation of these “positive” and “negative” overlapping genes showed enrichment in genes implicated in neurotransmission (whose products are localized in neurons, axons, dendrites and synapses) (FDR < 1e-4), and genes implicated in immune response (whose products are localized to lysosomes, lytic vacuoles, and phagocytic vesicles) (FDR < 1e-6), respectively (Supplementary Table8). Collectively, these findings suggest that 5-HT_2C_R editing in human and rat CNS is regulated via common pathways that at least in part converge at ADAR2.

### Association between Adarb1 and genome-wide gene expression

We then sought to determine whether the observed association between *ABCD* and global patterns of gene expression also held for the expression of *Adarb1* (the gene for ADAR2), under the premise that post-SCI changes in editing were likely to involve not only 5-HT_2C_R regulation but a larger network of ADAR2-related pathways. The frequency of *ABCD* was found to strongly correlate with *Adarb1* expression measured by qPCR (r = 0.743, p = 3.1e-4), and the qPCR measurement of *Adarb1* strongly correlated with its RNA-seq profile (r = 0.774, p = 6.8e-4). Analysis of the correlation between *Adarb1* qPCR measurements and genome-wide gene expression yielded 667 genes that were positively and 1027 genes that were negatively correlated with *Adarb1* (Supplementary Table9). Not surprisingly, given the strong correlation between *Adarb1* and *ABCD*, there was a substantial overlap between the sets of genes whose expression was positively or negatively associated with both *ABCD* and *Adarb1* (322 and 676 genes, respectively; hypergeometric p-values < 1e-100). Accordingly, functional annotation of the genes that were positively or negatively associated with *Adarb1* revealed significant enrichment for genes in similar categories that were detected for *ABCD* vs. gene expression analysis described above (Supplementary Table10).

Thus, we detected two large, substantially overlapping sets of genes for which expression was associated with the 5-HT_2C_R editing (*ABCD*) and/or *Adarb1* level. Within each set, the genes that were positively associated with *ABCD* or *Adarb1* showed enrichment for functional categories related to neurotransmission, whereas the negatively associated genes were enriched in categories related to immune response.

### Bayesian Network analysis

We then applied Bayesian network analysis (BNA), which reveals causal relationships among variables^36^, to identify genes that are “upstream” or “downstream” of *Adarb1*, using RNA-seq and qPCR *Adarb1* expression data obtained from SCI and control animals. The “upstream” genes might affect *Adarb1* expression (and consequently ADAR2*-*dependent mRNA editing); conversely, the “downstream” genes could be affected by *Adarb1* and editing. Among 14,638 analyzed genes, 10,071 showed no direct relationship with *Adarb1* and were classified as “independent”, 902 genes were identified as “upstream” and 2,001 genes as “downstream” of *Adarb1*, whereas 1,664 genes could not be assigned to any of these 3 categories and remained “ambiguous” (Supplementary Table11).

In an attempt to identify functionally relevant genes, we restricted further analysis to those “*Adarb1* upstream” and “*Adarb1* downstream” genes whose expression was significantly (FDR < 0.1) correlated with *Adarb1* expression (Supplementary Table10). Among the 902 “upstream” genes, 57 were positively and 270 were negatively correlated with *Adarb1* (hereinafter, “upstream positive” and “upstream negative” genes, respectively) (Supplementary Table12–13). Among the 2,001 genes “downstream” of *Adarb1*, 258 were positively and 305 were negatively correlated with *Adarb1* (hereinafter, “downstream positive” and “downstream negative” genes, respectively) (Supplementary Table14–15).

Functional annotation of these 4 sets of genes yielded information on the pathways that appear to be upstream or downstream of *Adarb1* expression (Supplementary Tables12-15). The “upstream positive” gene set was enriched in genes involved in the metabolism of cholesterol and other lipids as well as in categories related to transmission of nerve impulse and action potential. Along with *Adarb1*, many of these genes were down-regulated after SCI, e.g. oligodendrocyte-specific proteins (*Mag, Mbp*), proteins related to cholesterol biosynthesis (*Dhcr24, Fdft1d, Hmgcs1, Dhcr7*) and a potassium voltage gated channel, *Kcns3*. Altogether, 35 of the 57 “upstream positive” genes were down-regulated post-SCI, whereas none of them were up-regulated. Because myelin has the highest cholesterol content in the CNS^37^, these findings might reflect shutdown of lipid biosynthesis caused by the post-SCI oligodendrocyte death and demyelination resulting in increased concentration of free cholesterol as well as by the subsequent axonal conductance block^38^.

The “upstream negative” set was enriched in genes involved in immune response and its regulation. This finding reflects the long-lasting cellular and molecular inflammatory response induced by SCI^46^, which was exemplified by genes that encode neutrophil cytosolic factors (e.g., *Ncf1, Ncf4*), cytokines, cytokine receptors and related proteins (e.g., *Tnf, Tnfrsf9, Tnfrsf1b, Il16, Il10ra, Il4ra, Ifitm1, Irf8, Irf5, Ifi30, Ifi47, Ifngr1*), neurotrophic factors and their receptors (e.g., *Tgfbr1, Tgfb1, Ptafr),* matrix metalloproteinases (e.g., *Mmp19, Mmp3*), complement components and proteins that are involved in regulation of complement activation (e.g., *Cfh, C1r, C1s, C2, C1qc, C1qa, C1qb*), and other proteins that are expressed by activated microglia and monocyte-derived macrophages (e.g., *Cd86, Trem2, Tlr7, Tlr1, Tlr2)* and T lymphocytes (*Cd4*). The majority of the “upstream negative” genes (249 of 270), including the mentioned above, were up-regulated following SCI, and none of them were down-regulated.

The “downstream positive” set was enriched in genes involved in synaptic transmission and its regulation as well as synapse organization, and regulation of ion transmembrane transport. These categories are exemplified by genes that encode calcium channels (*Cacna2d2, Cacng2, Cacng7*), potassium channels (*Kcnab3, Kcnc1, Kcnc3, Kcnj12, Kcnj9, Kcnk10, Kcnt1, Hcn3*), glutamate receptors (*Grin2d, Grin3b*), adaptor protein in the postsynaptic density of excitatory synapses (*Shank1),* several guanine nucleotide-binding proteins (*Gnal, Gnao1, Gnaz, Gng7, Gng8*), and a voltage gated sodium channel (*Scn4b*). Expression of only 10 of the 258 “downstream positive” genes was altered post-SCI, and all these 10 genes (including *Kcnc3, Grin3b, Gnal, Scn4b, Panx2*) were down-regulated. Notably, *Grin3b* encodes a motoneuron-specific NMDA receptor modulatory subunit that reduces the NMDA conductance as well as calcium permeability^39^. The “downstream negative” set was enriched in genes involved in broad categories of response to wounding and wound healing, response to stress as well as in more specific categories of polysaccharide catabolism. Among the 305 “downstream negative” genes, 49 were up-regulated following SCI, and none were down-regulated. Interestingly, one of these 49 genes, *Maob*, encodes the mitochondrial monoamine oxidase which is found in neurons and astrocytes and catalyzes the oxidation of monoamines, including serotonin and epinephrine^40^. This finding suggests that the expression of *Maob* could be influenced by *Adarb1* levels.

## Discussion

In more than 80% of individuals with SCI, the injury is followed by development of debilitating spasms and spasticity in muscles innervated by spinal motorneurons below the site of injury^5^. Treatment of spasms with conventional antispastic drugs (e.g., baclofen) is often not efficacious or is not tolerated because of adverse side effects such as lethargy and weakness^41^. Post-SCI spasticity has multiple underlying mechanisms, and serotonin and its receptors play an important role in the etiology of this condition^42^. Recent reports suggest that 5-HT_2A_ and 5-HT_2C_ receptors on spinal motoneurons become constitutively active after SCI to compensate for the loss of brainstem serotonin, thus helping to recover motoneuron excitability and rudimentary locomotor function, yet contributing to muscle spasms^11^,^12^.

Whereas the post-SCI increase in 5-HT_2A_R constitutive activity has been reported to be achieved by up-regulation of its expression^12^, as also confirmed in this work, our recent study has presented evidence that the constitutive activity of 5-HT_*2C*_R was increased via alterations of mRNA editing^11^. Here we examined post-SCI changes of 5-HT_*2C*_R mRNA editing using MPS that yields numerous sequencing reads per editing region and therefore shows substantially increased precision and sensitivity^25^ compared to the cloning and sequencing method that was used in the initial study^11^. Although MPS revealed a pattern of the receptor variants that was significantly different from those reported previously^11^, the major findings of the initial study are corroborated in that the additive effect of the alterations we detected should be a substantially higher constitutive 5-HT_2C_R activity in the SCI rats. Most importantly, MPS demonstrated that the post-SCI decrease of 5-HT_*2C*_R editing involves only one of the 5 editing sites on the receptor mRNA, namely site D. Because site D is mostly edited by ADAR2, we predicted and indeed detected post-SCI down-regulation of ADAR2 but not ADAR1. Although correlation is not equal to causal relationship, it seems most likely that the decrease of ADAR2 activity in SCI rats indeed causes the drop in editing at site D.

We also demonstrated that, in addition to 5-HT_2C_R, SCI triggers editing changes in at least one more ADAR2 target, potassium voltage-gated channel K_v_1.1. This channel gives rise to a rapidly activated sustained outward current and plays an essential role in initiation and shaping of action potentials^43^. In spinal motoneurons, K_v_1.1 is localized mostly to axon initial segments, where it is critical for dampening neuronal excitability^44^. In addition to membrane potential, K_v_1.1 is regulated by its accessory inhibitory β subunit^45^. K_v_1.1 editing dramatically affects the binding of β subunit to K_v_1.1 protein, thus accelerating its recovery from inactivation at negative potentials and reducing the frequency of motoneuron firing^26^. Therefore, a post-SCI decrease in K_v_1.1 editing would enhance motoneuron excitability, which might contribute to spasms and spasticity in individuals with SCI. This prediction is in line with the earlier findings showing that the voltage threshold for the action potential is lower in motoneurons of chronic vs. acute spinal rats^45^ which could be, at least in part, explained by reduced Kv1.1 delayed rectifier current due to the decrease in Kv1.1 editing after SCI. Given the post-SCI down-regulation of ADAR2, it seems likely that in addition to 5-HT_2C_R and Kv1.1, SCI triggers editing changes in other receptors and/or channels which collectively contribute to the recovery of motoneurons and the concurring development of spasticity. Future work is expected to identify the entire spectrum of effects and the cellular specificity of ADAR2 down-regulation as well as its role in the pathophysiology of the SCI.

We detected numerous genes whose expression is altered 8 weeks after SCI. Analysis of these DE genes using WGCNA (which enables inference of the functional relevance of genes based on their network position^27^) revealed at least two distinct components. The first component appears to reflect transcriptional alterations associated with the profound and pervasive consequences of lesional inflammation caused by SCI, including activation and proliferation of resident microglia and astrocytes, infiltration of circulating innate immune cells (i.e., neutrophils, monocytes and lymphocytes), and enhanced intraspinal synthesis and release of cytokines, chemokines and other vasoactive substances (e.g., complement proteins) by neurons and non-neuronal cells^46^. This component shows enrichment for microglia- and astrocyte-specific genes, which are mostly up-regulated following SCI.

The second component apparently reflects post-SCI dysregulation of neurotransmission and lipid metabolism, and is represented by genes that are expressed in neurons or oligodendrocytes and are mostly down-regulated following SCI. Distinctly, the network analysis of the neurotransmission-related genes (see Fig. 5c) are compatible with the prediction that post-SCI adaptations of motoneurons are, at least in part, driven by the increased constitutive activity of 5-HT_2A/2C_Rs, which would enhance signaling through the PLC pathway^13^, thus mobilizing intracellular Ca^2+^, activating L-type Ca^2+^ channels and facilitating Ca^2+^ PICs^47^. The changes in the expression of lipid biosynthesis genes most likely are associated with the extensive oligodendrocyte cell death and demyelination after SCI.

**Figure 8:**
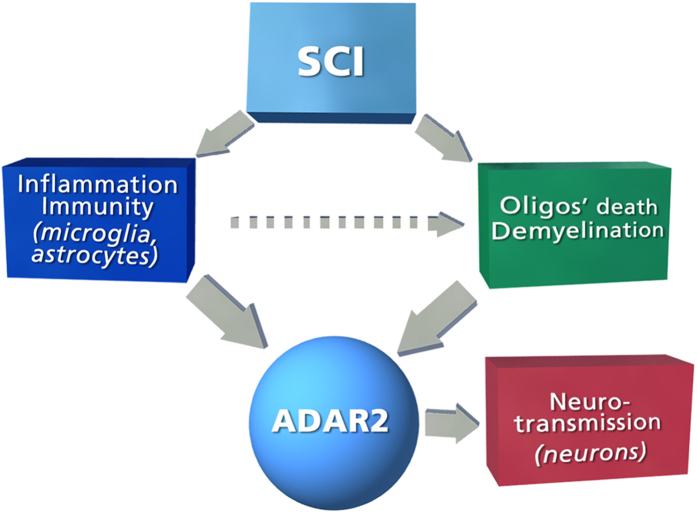
Schematic representation of the BNA results. SCI causes persistent inflammatory response which is maintained after 8 weeks in the spinal rats and includes activation and proliferation of resident microglia and astrocytes^46^. It also causes oligodendrocyte death and demyelination that are exacerbated by the inflammation^38^. Our BNA analysis suggests that SCI-induced down-regulation of the editing enzyme ADAR2 (and consequent decrease in editing of ADAR2 targets) is caused by these processes. BNA also suggests that post-SCI alterations in neuron-specific processes are downstream from ADAR2 and its targets. These downstream processes are likely to contribute to post-SCI recovery of motoneuron excitability but also to muscle spasms and spasticity.

We also detected numerous genes whose expression is positively or negatively associated with the level of 5-HT_2C_R editing in the rat spinal cord. The negatively correlated genes (which included *Adarb1*, but not *Adar*) are mostly related to inflammatory/immune response, whereas the positively correlated genes are mostly related to neurotransmission. These findings are fully compatible with the results of our recent 5-HT_2C_R editing study in the human PFC^35^. We also found a significant overlap between genes from the human and rat data sets that were positively or negatively associated with *ABCD* as well as significant overlap between genes that were correlated with both *ABCD* and *Adarb1* in the rat study. Taken together, these findings suggest that down-regulation of ADAR2 and the consequent decrease of 5-HT_2C_R and Kv1.1 editing are triggered by persistent post-SCI inflammatory response that is mediated by activation and proliferation of resident microglia and astrocytes and/or by post-SCI changes in neurotransmission which are associated with neurons and oligodendrocytes. In an attempt to distinguish between these mechanisms, we applied BNA that is designed to test for cause-and-effect relationships among variables. The BNA results strongly suggest that down-regulation of ADAR2 is mostly caused by inflammatory response triggered by SCI as well as by processes related to oligodendrocyte death and demyelination. The latter, at least in part, could be exacerbated by inflammation (Fig. 8). Notably, down-regulation of ADAR2 and the associated decrease of GluR2 editing is a profound pathological change that is relevant to the death of spinal cord motorneurons in amyotrophic lateral sclerosis (ALS)^48^. Although ALS had been once considered a motoneuron disease, it has been shown that motoneuron death is driven by a convergence of damaging mechanisms, including glial cell pathology and inflammatory conditions, such as microglial activation^49^. Oligodendrocyte dysfunction is also prevalent in ALS as indicated by gray matter demyelination that is observed in motor cortex and spinal cord of ALS patients^50^. Thus, similar mechanisms could be involved in down-regulation of ADAR2 in ALS and spinal cord injury. Furthermore, the association between ADAR2 and inflammation might provide a link to other CNS diseases (e.g., multiple sclerosis) in which inflammation is paralleled by spasticity.

BNA also indicates that post-SCI alterations in neuron-specific processes (e.g., synaptic transmission, synapse organization, and regulation of ion transmembrane transport) are mostly downstream of ADAR2 and consequently its editing targets. RNA editing, including ADAR2-dependent editing, is thought to act as a homeostatic mechanism that modulates the effect of a wide range of environmental and genetic factors that impair neuronal function^35^,^51^,^52^. Such a buffering function of ADAR2 is compatible with our observation that expression of many downstream genes is correlated with ADAR2 expression but independent of SCI. Conversely, SCI-associated changes in the expression of certain downstream genes (i.e., *Kcnc3, Grin3b, Gnal, Scn4b, Panx2, Vegf*), which are apparently mediated by the down-regulation of ADAR2 expression, likely reflect SCI-induced perturbations of the homeostasis. These downstream genes could belong to the network that contributes to post-SCI recovery of motoneuron excitability and related motor function but also to muscle spasms and spasticity. This network is apparently regulated by altered editing of ADAR2 targets (5-HT_2C_R, Kv1.1, and possibly, others), and characterization of this network is a goal of our future work.

## Conclusions

In this study we present evidence that the decrease of 5-HT_2C_R editing after SCI is most likely mediated by down-regulation of the editing enzyme ADAR2. We additionally found that SCI causes a substantial decrease in the mRNA editing level of at least one more ADAR2 target, Kv1.1 channel. Bioinformatic analysis of editing and genome-wide transcriptome data indicates that down-regulation of ADAR2 and the ensuing decrease of editing in its targets is triggered by the persistent inflammatory response to SCI that is manifest in a long-term activation of microglia. The results of this analysis further suggest that down-regulation of ADAR2 after SCI results in significant changes of the expression of multiple genes in neurons. The known functions of the affected genes implicate them in the restoration of neuronal excitability that is also associated with post-SCI spasms, a finding that could have broad implications for other diseases and injuries of the nervous system. The results of this work start to elucidate the specific molecular mechanisms that are associated with alterations of RNA editing in SCI and ultimately could lead to effective anti-spasticity treatments.

## Methods

### Sacral spinal injury model in rat and relation to human spasticity

In our sacral spinal injury model, only tails (not bladder or hindlimbs) are affected in injured animals^5^. Previously, we have shown that the spasticity that emerges in the tail of chronic spinal rats closely mimics the human spasticity syndrome seen in the legs after SCI, with clonus, hypertonus, hyperreflexia, spasms, and general delayed onset of symptoms^5^. In particular, flexor spasms emerge first, followed by extensor spasm (months), as also seen in humans. Finally, we have recently shown that the same ionic mechanism that mediates spasms in the tail of rats also mediates spasms in humans (PICs in motoneuron)^9^, thus justifying the use of the sacral spinal rat as a model of SCI-induced spasticity in humans.

In the current study we used both normal and spastic (with chronic spinal cord injury) female Sprague-Dawley rats. For the spastic rat, a complete spinal cord transection was made at the S2 sacral level when the rats were 7–8 weeks old, as previously described^5^. Briefly, under general anesthetic (sodium pentobarbital, 58.5 mg·kg^–1^) and sterile conditions, a laminectomy was performed on the L2 vertebrae to expose the S2 spinal cord. The dura was slit transversely, and 0.1–0.3 ml Xylocaine (1%) was applied topically. Under a surgical microscope, the spinal cord was transected by holding the pia with forceps and sucking under the pia with a fine suction tip. Caution was needed to avoid damaging the anterior artery or posterior/dorsal vein, since the sacrocaudal spinal cord dies without this midline vasculature. The dura was closed with two 8-0 silk sutures, and the muscle layers and skin were tightly sutured over the cord, and the rat allowed to recover. Usually within 30 days dramatic spasticity in the tail muscles (which are innervated by sacrocaudal motoneurons below the level of the injury) was developed in the injured rats. Only animals with clear signs of spasticity were included in the study (see^5^ for details of spasticity assessment).

Spinal rats were euthanized 8 weeks post injury and control animals were euthanized when they were 15 weeks old using urethane (0.18 mg per 100 g). The lumbosacral spinal cord was then exposed by scissor laminectomy, excised and transferred to a dissection dish containing RNA stabilizer (RNAlater, Life Technologies) for removal of roots and blood. The cord extraction procedure was limited to ~ 5min in order to preserve the integrity of RNA. To further minimize possible RNase contamination, all tools, dishes, and gloves were autoclaved and treated with RNaseZap (Life Technologies). Finally, the cords were cut into lumbar and sacral sections, placed in Eppendorf tubes, frozen in liquid nitrogen, and stored at 75 °C. All animal use and procedures were performed in accordance with Canadian Council for Animal Care guidelines, and all experimental protocols were approved by the University of Alberta animal welfare committee

### RNA extraction and cDNA synthesis

Total RNA was extracted using Invitrogen ToTally RNA Kit (Life Technologies) that includes two rounds of phenol-based extractions and DNase treatment Only samples that yielded high quality RNA [RNA integrity number (RIN) ≥ 8.0] by 2100 Bioanalyzer (Agilent)] were used. The average RIN for the specimens was 8.73 ± 0.09 (Mean ± SEM). cDNA was synthesized from equal quantities of RNA from each animal (500 ng of RNA per 10 μl of reaction) using High Capacity cDNA Reverse Transcription kit (Life Technologies).

### Analysis of 5-HT_2C_R editing

5-HT_2C_R RNA editing was assessed using massively parallel sequencing (MPS) of PCR-amplified DNA fragments in the region of editing as previously described^23^,^35^. Because inosine is read as guanine during reverse transcription, MPS provided the number of reads for each of the 32 5-HT_2C_R mRNA editing variants in each spinal cord sample. The relative frequency of each variant in each sample was calculated as the ratio of the number of reads detected for a particular variant to the total number of reads for all variants in this sample.

***Adarb1* and *Adar* mRNA expression** was measured by real time quantitative PCR (qPCR) using an ABI 7900 thermocycler and TaqMan gene-specific assays (Life Technologies) as described^53^. Expression of the target transcripts were normalized to the geometric mean of three endogenous control genes (ECG): Glyceraldehyde 3-phosphate dehydrogenase *(Gapdh),* beta-actin *(Actb),* and cyclophilin A (*Ppia)*. The relative expression of the target transcripts and ECGs were determined using the Relative Standard Curve Method, which provides accurate quantitative results by accounting for differences in the efficiencies between target and ECG amplifications^53^.

### Analysis of Kcna1 editing

To assess the level of *Kcna1* editing, we employed a modified *Mfe1* restriction assay that is commonly used for measuring mRNA editing when it occurs at a single site^26^. *Kcna1* editing results in re-coding from Ile to Val. The editing event destroys an *MfeI* restriction enzyme recognition site. A constitutive *MfeI* site is also found 50 bp upstream of the editing site. Amplification products resulting from RT-PCR digested with *MfeI* will give two products differing by 50 bp. Specifically, we used forward CACTCCAAGGGCCTTCAGATCCTG and reverse CTGTCAGAGGCTAAGTTAGGAGAACTAACA primer pair which results in 390bp amplicon. Digestion of the “unedited” amplicon with *Mfe1* generates 220 bp, 51 bp, and 119 bp DNA fragments, whereas digestion of “edited” amplicon yields only two fragments (220 bp and 170 bp). For each spinal cord sample quantified, two independent PCRs were carried out each time in duplicate. The resulting amplicon was cut with *MfeI* and analyzed using Bioanalyzer 2100 (Agilent). The ratio between the 171 bp and 120 bp fragments defined the efficiency of *Kcna1* editing.

### Iba-1 Immunohistochemistry

SCI and control rats were euthanized with a pentobarbital overdose (at 240 mg/kg) 8 weeks post-SCI. Spinal cords were dissected, post-fixed in 4% paraformaldehyde for 24 hrs, and cryoprotected in 30% sucrose in 0.1 M phosphate buffer for 48 hrs. Then, lumbosacral spinal cord segments were embedded in Tissue Tek (Sakura Finetek), mounted onto filter paper and frozen at −40 °C in 2-methylbutane (Fisher Scientific). Longitudinal tissue sections containing the lesion were cut at 25 μm in a Cryostar NX70 (Fisher Scientific) and staggered across four sets. The slides were stored at −20 °C until further processing. For the labelling, slides were warmed at 34 °C for 1 hr and rehydrated 3 × 10 min in TBS, which was followed by 1 hr blocking in 10% NGS in 0.5% TBS-TX at room temperature. Next, the tissue was incubated with the primary antibody (rabbit anti-iba-1; 1:1000; Wako) in 1% NGS and 0.5% TBS-TX overnight at 4 °C. The tissue was then washed 3 × 10 min in 0.5% TBS-TX and incubated with the secondary antibody (AF488 goat anti-rb; 1:400; Molecular Probes) for 2 hrs at room temperature in 1% NGS and 0.5% TBS-TX. Finally, slides were washed 2 × 10 min in 0.5% TBS-TX and 2 × 10 min in TBS, and cover-slipped wet with Fluoromount G (SouthernBiotech). The images were obtained using a Leica DM6000 B fluorescence microscope. Number of Iba-1 positive cells in the central grey matter was counted at 200x magnification. Images were opened on ImageJ and cells counted with the cell counter plugin. Criterion for counting included sharp focus and the presence of clear (round or oval) cell bodies.

### RNA-seq

RNA-seq uses MPS to allow transcriptome analyses of genomes at a far higher resolution that is available with Sanger sequencing or microarray-based methods. RNA-seq involves direct sequencing of complementary DNA generated from the RNA of interest using next-generation sequencing technologies. The obtained reads are then aligned to a reference genome in order to construct a whole-genome transcriptome map.

Total RNA (1 μg) extracted from spinal cord specimens (see above) was processed using the TruSeq RNA library preparation kit V2 (Illumina) (which generates mRNA-focused libraries from total RNA) according to manufacturer’s instructions. The libraries were sequenced using an Illumina HiSeq2000. After de-multiplexing of each library pool according to its barcode sequence, 27.5 × 10^6 ^± 6.4 × 10^6^ (MEAN ± SEM) total reads per sample were available for analysis. Alignment, assembly and quantification of the data were performed using TopHat^54^ against rat genome version rn4 obtained from UCSC browser.

### Gene Expression Analysis

The numbers of reads for each gene were obtained using the aligned reads generated by TopHat. Among the 17,233 refseq gene transcripts annotated for the rat genome (version rn4), 1,985 transcripts were removed, maintaining only those with at least one read in at least one sample. This resulted in a total of 15,248 transcripts. For each gene that was represented by several transcripts, only one transcript that resulted in a signal with the highest SD was retained. After this filtering, the data for 14,638 unique gene symbols were retained, and those genes were used in subsequent analyses. The counts data are normalized by the function varianceStabilizingTransformation in the Bioconductor DESeq package^55^.

### Differential Expression Analysis

RNA-Seq data were compared between SCI vs. control rats using the Bioconductor DESeq package^55^. The differentially expressed (DE) genes were defined as those with fold change (SCI vs. controls) of >1.2 or <0.8 (for positively and negatively DE genes, respectively) and false discovery rate (FDR) adjusted p values < 0.05.

### Weighted gene co-expression analysis (WGCNA)

Gene coexpression relationships among the DE genes were analyzed by WGCNA, and the co-expression modules were identified. WGCNA begins with a matrix of the Pearson correlations between all gene pairs, then converts the correlation matrix into an adjacency matrix using a power function f(x) = x^β. The parameter β of the power function is determined in such a way that the resulting adjacency matrix (i.e., the weighted co-expression network), is approximately scale-free. To explore the modular structures of the co-expression network, the adjacency matrix is further transformed into a topological overlap matrix for module detection using a dynamic cut-tree^27^. To distinguish between modules, each module is assigned a unique color identifier, with the remaining, poorly connected genes colored grey.

Functional enrichment for genes within the identified coexpression modules was assessed using the GeneGo MetaCore software.

### Correlational and overlap analyses

Spearman correlation test was used to test the correlations between *ABCD* frequencies as quantified by MPS (or *Adarb1* as quantified by qPCR) and an expression profile of each gene detected by RNA-seq. Multiple testing adjustment was performed using the Benjamini and Hochberg method.

Significance of the overlap between the *ABCD* signature genes in the rat spinal cord developed in this study and the *ABCD* signature in the human PFC^35^ was assessed using Fisher exact test, separately for the positively and negatively correlated genes. First, we identified symbols of gene detected in both studies; this yielded 11,997 gene symbols. From this list of genes, we built the contingency table counting the genes associated with *ABCD* in both studies, and performed Fisher exact test using this table.

### Bayesian Network analysis (BNA)

A Bayesian Network consists of a graphical structure and a probabilistic description of the relationships among variables in a system^56^. The graphical structure explicitly represents cause-and-effect assumptions that allow a complex causal chain linking actions to outcomes to be factored into an articulated series of conditional relationships. Each of these relationships can then be independently quantified using a sub-model suitable for the type and scale of information available.

BNA of the relationship between genome-wide gene expression (measured by RNA-seq) and *Adarb1* (measured by qPCR) was performed using the ‘deal’ R package (http://www.R-project.org/. The genes were analyzed one at a time. For each gene, an analysis was conducted including the following variables: ‘treatment’ (discrete: SCI vs. control), “gene expression” (continuous: normalized read counts), and “*Adarb1*” (continuous: qPCR quantification). For each gene, there were 2 possible relationships (links) between the treatment (SCI) and the expression of this gene (no link or a link directed from the treatment to gene expression but not vice-versa), and there were 3 possible links between *Adarb1* and the expression of this gene (no link, a link directed from a gene to *Adarb1,* and a link directed from *Adarb1* to a gene), resulting in a total of 12 possible networks (3 possible networks between a gene and *Adarb1 *× 2 possible networks between a gene and the treatment    × 2 possible networks between *Adarb1* and the treatment). Based on this, for each gene we estimated 12 possible networks and ranked then according to their score. Then, we categorized each gene according to its relationship with *Adarb1* based on the following criteria:

1. if the relative score of the second best network compared to the first was above 0.9, a gene was classified as “ambiguous”;
2. if the top network showed an outgoing edge from a gene to *Adarb1*, a gene was classified as “upstream”;
3. if the top network showed an outgoing edge from *Adarb1* to the gene, a gene was classified as “downstream”;
4. if none of the above 3 conditions were met, a gene was classified as “independent” (with regard to *Adarb1*).

**Figure 9:**
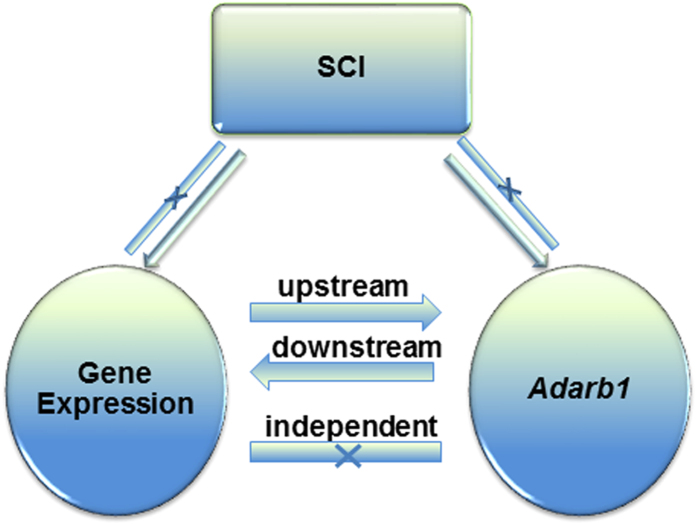
Bayesian Network Analysis (BNA). For each expressed gene, prior knowledge indicates 12 possible arrangements of causal links between treatment (SCI), gene expression of each gene, and *Adarb1*. By performing BNA, we can define the relationship between each gene and *Adarb1* by placing it within one of 4 distinct categories: upstream downstream, independent, and ambiguous (genes for which the distinction is unclear). “X “depicts the absence of a link.

A schematic of the workflow is presented in Fig. 9.

## Additional Information

**How to cite this article**: Narzo, A. F. D. *et al*. Decrease of mRNA Editing after Spinal Cord Injury is Caused by Down-regulation of ADAR2 that is Triggered by Inflammatory Response. *Sci. Rep*. **5**, 12615; doi: 10.1038/srep12615 (2015).

## Supplementary Material

Supplementary Table 1

Supplementary Table 2

Supplementary Table 3

Supplementary Table 4

Supplementary Table 5

Supplementary Table 6

Supplementary Table 7

Supplementary Table 8

Supplementary Table 9

Supplementary Table 10

Supplementary Table 11

Supplementary Table 12

Supplementary Table 13

Supplementary Table 14

Supplementary Table 15
